# Survey participation among general practitioners: comparison between teaching physicians and a random sample

**DOI:** 10.1186/s13104-021-05895-z

**Published:** 2022-01-10

**Authors:** Michael Pentzek, Verena Baumgart, Flora-Marie Hegerath

**Affiliations:** 1 Institute of General Practice (ifam), Centre for Health and Society (chs), Medical Faculty, Heinrich Heine University, Moorenstr. 5, Building 17.11, 40225 Düsseldorf, Germany; 2 Department of Applied Health Sciences (DAG), Hochschule für Gesundheit, University of Applied Sciences, Gesundheitscampus 6-8, 44801 Bochum, Germany; 3 Department of General Practice and Family Medicine, Faculty of Medicine, Ruhr University Bochum (RUB), MAFO 01/256, 44780 Bochum, Germany

**Keywords:** Recruitment, General practice, Research participation, Response rate, Postal survey, Online survey, Teaching physician

## Abstract

**Objective:**

Health scientists strive for a smooth recruitment of physicians for research projects like surveys. Teaching physicians are an easy to approach population that is already affiliated with a university by teaching students in their practice. How do response rates compare between a convenient online survey among teaching physicians and an elaborate postal survey in a random sample of unknown physicians? Data from the TMI-GP study on the use of memory tests in general practice were used.

**Results:**

Physicians in the random sample responded to the postal survey more often than teaching physicians to the online survey (59.5% vs. 18.9%; odds ratio 7.06; 95% confidence interval 4.81–10.37; p < 0.001). Although it is unclear whether the sample, the survey mode (online vs. postal) or both account for this effect, it is noteworthy that even in such a convenience sample of known/committed physicians, an adequate response rate could not be reached without a tailored and elaborated survey technique. Responders in the two samples were comparable regarding a content-related item (use of memory tests; Χ^2^ (df = 1) = 3.07; p = 0.080).

**Supplementary Information:**

The online version contains supplementary material available at 10.1186/s13104-021-05895-z.

## Introduction

Surveys are an important research method in health sciences, but poor participation of physicians is an international problem [1–3]. Concerning the survey mode, it has been reported, that general practitioners (GPs) and other health care professionals prefer postal over online/email surveys, and mixed method approaches are recommended [4].

All over the world, practicing GPs teach medical students in the discipline of general practice/family medicine in their offices [5–7]. This teaching activity is usually appreciated with a small monetary incentive (estimated 100–200 Euro per week per student in Germany). GP trainers (alias teaching physicians) are in regular contact with a university and could therefore perhaps be recruited for research projects with less effort. Especially in the absence of robust GP-based research networks, university institutes resort to their own teaching GPs for research purposes. The advantages of recruiting teaching GPs for research include the convenience of using routines for contacting (established modes and latest addresses), a personal connection and commitment of GPs to the university, and no need for approaching new GPs via address lists from agencies or authorities. Overall, the expectation is that recruiting teaching GPs for research will lead to high participation at little expense. Recruitment of GPs from random samples (without any link to university and/or research) requires more time and economic resources to reach acceptable participation rates [8]. The advantages of random samples include more generalizable and less biased results. Consequently, for researchers it is important to know if low-effort recruitment of teaching GPs and high-effort recruitment of random GPs gain different participation rates. We compared these two approaches in a GP survey with two separate samples: a sample of teaching GPs with convenient contacting by fax or mail using an efficient online survey mode vs. a random sample with an elaborated postal strategy using paper–pencil questionnaires. Data come from a study on the use of tests for memory impairment in general practice (TMI-GP study), itself a complement to the SMI-GP study (subjective memory impairment in general practice) [9]. The TMI-GP survey has not yet been published; an English translation of the questionnaire is attached (see Additional file 1). For the TMI-GP study, the adjunct sample of teaching GPs was used to boost the sample size at lower cost and to obtain appropriate analysis conditions.

## Main text

### Samples

A gender-stratified random sample of 400 GPs was drawn from the database of the Association of Statutory Health Insurance Physicians in the North Rhine Region (KVNO). For the teaching GP sample, four German university institutes of general practice identified all of their teaching GPs in practice: n = 76 Bochum, n = 287 Düsseldorf, n = 140 Münster, n = 86 Rostock.

### Questionnaire

In the SMI-GP and TMI-GP studies, a questionnaire on the use of memory tests in general practice was constructed as part of a sequential exploratory mixed methods design [10]. Question wording and scaling were in accordance with scientific standards [11] and additionally aided by cognitive interviews [12]. The final questionnaire comprised 11 topics and 68 items (four pages in its paper version).

### Surveys

Both surveys were anonymous, dispatched in early December 2019 and designed according to best practices for invitation, layout, item construction, questionnaire design and reminder strategy [13, 14]. The invitation letter/fax/email was personalised (e.g., individual salutation, photo of the researcher). A reminder letter/fax/email was send in late January 2020, accompanied by a copy of/link to the (paper/online) questionnaire.

### Differences between postal and online survey

Dispatch of the postal survey for the random sample GPs was done with coloured envelopes, high-quality paper, handwritten addressing and stamps. A non-monetary unconditional incentive (pen with a “Many thanks!” print) and an addressed return envelope with stamps were enclosed. Dispatch of the online survey for the teaching GPs was done by employees of the four institutes on behalf of the investigators (“We would like to invite you to a survey of our partner institute of general practice in Düsseldorf […] The survey is conducted by student Flora-Marie Hegerath and accompanied by PD Dr. Michael Pentzek.”). The mode of recruitment was chosen by the institutes according to the way each GP is usually approached by the institute for other purposes (by e-mail 73.5%, by fax 26.5%). No incentivisation was offered. The links in emails and faxes led to the online questionnaire, which was realised on the online survey platform SoSci Survey [15].

### Analyses

A returned questionnaire with ≥ 90% item response was considered as participation. The multivariable influence of sample (teaching/random), GP gender and the interaction of both on response rate (response/non-response) was calculated by binary logistic regression. A content-related comparison of reponders from both samples was made on a central item (use of memory tests yes/no) performing a chi-square test.

### Results

Table 1 shows the characteristics of both samples.

**Table 1:** Description of the two general practitioner samples

	Teaching GP sample (online survey)	Random GP sample (postal survey)	Total
N contacted			
Total	589	400	989
Women	210 (35.7%)	200 (50.0%)	410 (41.5%)
Men	379 (64.3%)	200 (50.0%)	579 (58.5%)
N non eligible^a^	6	2	8
N analysed sample	583	398	981
N returned questionnaires	120 (20.6%)	269 (67.6%)	389 (39.7%)
N < 90% item response (valued as non-response)	10 (1.7%)	32 (8.0%)	42 (4.3%)
N valid response	110 (18.9%)	237 (59.5%)	347 (35.4%)
Experience as GP [years in practice, mean (SD)]	18.7 (8.7)	17.7 (9.7)	18.1 (9.4)
Using memory tests in practice			
N yes	97 (88.2%)	191 (80.6%)	288 (83.0%)
N no	13 (11.8%)	46 (19.4%)	59 (17.0%)

*GP* general practitioner, *N* number, *SD* standard deviation

^a^Termination of practice, parental leave, incorrect contact information

On one of the central survey items (use of memory tests yes/no), teaching sample GPs and random sample GPs do not differ from each other [Χ^2^ (df = 1) = 3.07; p = 0.080].

**Fig. 1:**
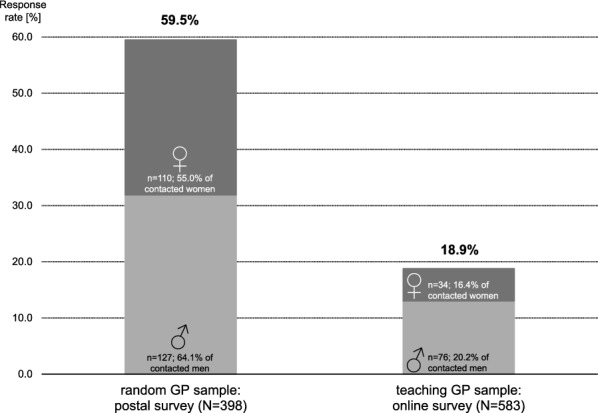
Response rates of male and female GPs in two samples. *GP* general practitioner

Teaching GPs responded to the online survey less often than the GPs in the random sample to the postal survey (see Fig. 1). In the logistic regression analysis this effect of sample on response rate (Odds ratio OR 7.06; 95% confidence interval CI 4.81–10.37; p < 0.001) is independent from the effects of gender (0.78; 95% CI 0.50–1.21; p = 0.264) and the interaction term gender*sample (OR 0.88; 95% CI 0.48–1.61; p = 0.679).

### Discussion

A convenient, inexpensive and time-saving online survey strategy in a sample of university-affiliated teaching physicians is by far not reaching the response rate of an elaborated postal survey strategy in a random physician sample. This finding, especially to this extent of the difference in response rates (40%), is surprising and requires a discussion of possible causes.

In this study, we compared not only two different samples, but also two different survey strategies in their entirety– one convenient (known GPs, fax/email invitation, online survey tool, no incentives) and one elaborate (unknown GPs, postal dispatch/return with stamps, paper questionnaires, incentives). Thus, our finding can be attributed to the effect of the sample, the effect of the method, or a combination of both (the strategies as a whole).

A sample effect would have been expected in the other direction: Compared with random GP samples, teaching GP samples reportedly yield significantly higher response rates [16]. One substantial reason is the existing affiliation with the research institute and researchers [17]. In our study, the latter effect may have been blunted: Although the teaching GPs were recruited by their affiliated institutes, in three institutes this recruitment was done "on behalf" of another institute. This may have weakened the personal link between GP and researcher. Another possible reason for the lower response rate in the sample of teaching physicians reported here could be the existing workload for student teaching, reducing the commitment for other university-related activities such as research. The importance of work-life balance and a missing incentive for survey participation (opposed to a remuneration of teaching, see Introduction section) may have strengthened this effect.

The survey method may have had an impact on response rates. It is well known that GPs and other health care professionals prefer postal over online/email surveys [13, 18]. The exact reasons for this have not yet been sufficiently investigated [19], which is why the following factors are hypothetically based on unsystematic feedback and personal research experience: Access problems or data protection reasons may hinder the GP to access the online questionnaire from his/her office computer. A general aversion to online surveys may have had an impact (e.g., caused by too many requests or experiences with unserious providers). Invitation by email or fax in the teaching GP sample (vs. letter in the random sample) may have attenuated the response rate: The transfer of the link from fax could have been a barrier. Emails to the practice address may not be read regularly and/or may be read by assistants rather than the GP him/herself. Faxes and emails may not attract much attention because they are no different from the many other faxes and emails that reach the practice every day. The possibilities of personalisation, appreciation, attracting attention, incentivisation etc. can be implemented better or more directly and tangibly with postal surveys (especially with regard to materials such as high-quality paper, stamps, coloured envelopes, handwritten addressing, gifts like pens etc.). These features may attract more attention than a fax or an e-mail with few distinctive attributes.

### Conclusion

Method beats sample. Even in a convenience sample of known/committed physicians, adequate response rates could not be reached without a tailored and elaborated survey technique.

## Limitations

In this study, we did not apply a factorial experimental design with distinct varying factors. As we compared two different complex strategies, we cannot differentiate the impact of single survey features (e.g., sample, invitation, mode, incentive) on response rate. Remaining open questions are grounded in this flaw and in how the method may have interacted with the kind of sample to influence response rates. Would the teaching GPs have shown higher response rates when approached with a postal survey? Would the random GPs have shown lower response rates when invited by email or fax? What exactly was the problem with the online survey in the teaching GP sample? What effect would other modes of invitation and survey (e.g., per SMS, messenger) have had on participation? Would different forms of incentives (e.g., donations for social/environmental charity, vouchers, online money transfer) have enhanced the response rate in the online survey?

In concordance with earlier research [16], in the present study the samples of random and teaching GPs were comparable at the content level, which argues in favour of being able to merge samples and analyse them together at the content level. However, this was verified for only one item and in only < 20% of teaching GPs (with high interest in the survey topic?). More complex analyses of GP sample comparability are important to consider in future research.

Research in general practice uses manifold qualitative, quantitative and mixed methods designs. Our results are limited to the participation in a survey. No conclusions about participation of different GP samples in other study designs can be drawn.

We did not conduct a non-response analysis. Possible differences between responders and non-responders in both samples would have yielded further important information. We also missed to ask GPs for their explicit reasons for participation and non-participation.

## Supplementary Information

**Additional file 1. **TMI-GP questionnaire (Tests for memory impairment in general practice). Items—English translation of German original.

## Data Availability

The dataset used and analysed during the current study is available from the corresponding author on reasonable request.

## Abbreviations

GP: General practitioner
TMI-GP: Study on tests for memory impairment in general practice
SMI-GP: Study on subjective memory impairment in general practice
N: Number
SD: Standard deviation
OR: Odds ratio
CI: Confidence interval
