# Supplementary material for: Survey participation among general practitioners: comparison between teaching physicians and a random sample
Source: BMC Res Notes. 2022 Jan 10;15:9. doi: 10.1186/s13104-021-05895-z (PMC8751373; doi:10.1186/s13104-021-05895-z)
Supplement: Supplementary file 1 — Additional file 1. TMI-GP questionnaire (Tests for memory impairment in general practice). Items—English translation of German original. [file 13104_2021_5895_MOESM1_ESM.pdf]

TMI-GP questionnaire (Tests for memory impairment in general practice)
Items – English translation of German original

Below you can see some **statements from other GPs on the subject of memory tests**. Please indicate to what extent you **agree with** these statements.

(1 cross per line!)	*not at all*	*rather not*	*rather*	*entirely*
"Memory tests disrupt doctor-patient communication".	❑	❑	❑	❑
"Memory tests can be integrated into our everyday practice."	❑	❑	❑	❑
"Memory tests have a benefit for patients."	❑	❑	❑	❑
"Memory tests give me as a GP additional diagnostic information."	❑	❑	❑	❑
"The memory tests available are an imposition for patients."	❑	❑	❑	❑
"Memory tests are primarily the responsibility of neurologists/psychiatrists. “	❑	❑	❑	❑
"Memory tests are adequately remunerated."	❑	❑	❑	❑

How do you clarify **memory problems** when you or the patient have an initial suspicion?

(1 cross per line!)	*never/ex-ceptionally*	*some-times*	*frequently*	*always*
Referral for clarification	❑	❑	❑	❑
In conversation with individual questions (e.g. "*What did you eat yesterday?* "or similar)	❑	❑	❑	❑
With a memory test here in practice	❑	❑	❑	❑
The patient's self-perception of his memory is asked about	❑	❑	❑	❑
In a basic geriatric assessment	❑	❑	❑	❑
By means of external anamnesis (if patient agrees)	❑	❑	❑	❑

Are standardised **memory tests carried out on your** patients in your practice?

❑ No

❑ Yes

If memory tests are carried out, **who conducts memory tests in your practice**?

(1 cross per line!)	*never*	*some-times*	*frequently*	*always*
Test them yourself	❑	❑	❑	❑
A medical practice colleague tests	❑	❑	❑	❑
Practice nurse or other practice staff tests	❑	❑	❑	❑

If your patients are **not (any longer) tested in** your practice - are there reasons for this?

....

....

....

....

If memory tests are generally carried out on your patients in your practice (no matter by whom), I would ask you to answer the following questions.

The following is about performing memory tests on all your patients aged 65 and over.

Which memory tests are performed on your patients in your practice and how often?
Please roughly estimate the **frequency per quarter.**

How often per quarter? (1 cross per line!)	*never*	*1 - 3*	*4 - 7*	*8 - 12*	*13 - 20*	*21 - 30*	*> 30*
**MMSE** (Mini Mental State Examination)	❑	❑	❑	❑	❑	❑	❑
**VAT** (Visual Association Test)	❑	❑	❑	❑	❑	❑	❑
**MoCA** (Montreal Cog. Assessment)	❑	❑	❑	❑	❑	❑	❑
**SKT** (Short Cognitive Performance Test)	❑	❑	❑	❑	❑	❑	❑
**Clock drawing test**	❑	❑	❑	❑	❑	❑	❑
**DemTect** (dementia detection test)	❑	❑	❑	❑	❑	❑	❑
**TE4D** (Test for the early detection of dementia with discrimination from depression)	❑	❑	❑	❑	❑	❑	❑
Other : ____________________	❑	❑	❑	❑	❑	❑	❑

Please rate **how important the** following **reasons for taking memory tests** are to you.				
(1 cross per line!)	***not at all impor-tant***	***less impor-tant***	***Impor-tant***	***very impor-tant***
Differentiation between depression and dementia	❑	❑	❑	❑
Early detection of mild dementia	❑	❑	❑	❑
Repeated testing to detect early memory changes (deterioration in score)	❑	❑	❑	❑
To decide whether to refer or not	❑	❑	❑	❑
Assurance/documentation that memory has been checked	❑	❑	❑	❑
Deriving concrete next steps from the test result	❑	❑	❑	❑
Mapping the course of the disease (progress monitoring)	❑	❑	❑	❑
Measuring therapy effects	❑	❑	❑	❑
Mapping the severity of dementia	❑	❑	❑	❑
Patient wishes a test	❑	❑	❑	❑
Reassuring worried patients	❑	❑	❑	❑
Reassurance of relatives	❑	❑	❑	❑
To offer anything at all when dementia is suspected	❑	❑	❑	❑

How are **the test situation and the discussion of results arranged**?				
(1 cross per line!)	***no***	***rather not***	***rather***	***yes***
The relatives are present during the testing.	❑	❑	❑	❑
If there is time in the practice, testing is done directly (without a separate appointment).	❑	❑	❑	❑
The patient is told whether the test result speaks for or against a suspicion of dementia.	❑	❑	❑	❑
The discussion of the test result is charged with a code.	❑	❑	❑	❑

The following two questions are about your personal attitude towards memory tests (no matter if you do memory tests yourself or not).

How important are the following aspects for you to suspect dementia for the first time?

(1 cross per line!)	*not at all*	*not*	*rather not*	*rather*	*very*	*extre-mely*
Your own gut feeling	❑	❑	❑	❑	❑	❑
Memory test score	❑	❑	❑	❑	❑	❑
Age of the patient	❑	❑	❑	❑	❑	❑
Vascular risk factors	❑	❑	❑	❑	❑	❑
Positive family history of dementia	❑	❑	❑	❑	❑	❑
Complaints of the patient regarding his memory	❑	❑	❑	❑	❑	❑
Indications from medical colleagues	❑	❑	❑	❑	❑	❑
Indications from relatives/environment	❑	❑	❑	❑	❑	❑
Overall impression of the patient	❑	❑	❑	❑	❑	❑
Indications from your practice staff	❑	❑	❑	❑	❑	❑
Changes in score between two memory tests	❑	❑	❑	❑	❑	❑
Laboratory parameters	❑	❑	❑	❑	❑	❑

Below you can see some **statements from other GPs on the subject of memory tests**.
Please indicate to what extent you **agree with** these statements.

(1 cross per line!)	*not at all*	*rather not*	*rather*	*entirely*	*not ass-essable*
"I'm uncomfortable with test taking".	❑	❑	❑	❑	❍
"The interpretation of test result is difficult for me."	❑	❑	❑	❑	❍
"Patients participate well on memory tests."	❑	❑	❑	❑	❍
"When interpreting the test result, the cut-off value is crucial. "	❑	❑	❑	❑	❍
"Informing about test results is unpleasant."	❑	❑	❑	❑	❍
"A positive test result has diagnostic and/or therapeutic consequences for me. "	❑	❑	❑	❑	❍
"For patients with problems in speech, hearing or vision, the test result is more difficult to interpret."	❑	❑	❑	❑	❍
"Since memory tests can be billed, we do them more often."	❑	❑	❑	❑	❍
"The patient's behaviour in the test situation is more informative for me than the score."	❑	❑	❑	❑	❍
